# Advancing Lead Exposure Studies in Remote Settings: Method Development and Application of Lead Stable Isotope Analysis in Dried Blood Spots from Suriname, South America

**DOI:** 10.3390/toxics14080715

**Published:** 2026-08-13

**Authors:** Manasi Simhan, Sean R. Scott, Martin M. Shafer, Jenny N. Poynter, Gaitree K. Baldewsingh, Wilco C. W. R. Zijlmans, Anisma R. Gokoel, Maureen Y. Lichtveld, Jeffrey K. Wickliffe, Shannon M. Sullivan

**Affiliations:** 1 Nelson Institute for Environmental Studies, University of Wisconsin-Madison, Madison, WI 53707, USA; 2 Wisconsin State Laboratory of Hygiene, School of Medicine & Public Health, University of Wisconsin-Madison, Madison, WI 53718, USAmmshafer@wisc.edu (M.M.S.); 3 Department of Pediatrics, University of Minnesota, Minneapolis, MN 55455, USA; poynter@umn.edu; 4 Medical Mission Primary Health Care Suriname, Paramaribo, Suriname; gbaldewsingh@medischezending.sr; 5 Foundation for Perinatal Interventions and Research in Suriname, Paramaribo, Suriname; wilco.zijlmans@uvs.edu; 6 Faculty of Medical Sciences, Anton de Kom University of Suriname, Paramaribo, Suriname; anishmag@gmail.com; 7 School of Public Health, University of Pittsburgh, Pittsburgh, PA 15267, USA; mlichtve@pitt.edu; 8 School of Public Health, Department of Environmental Health Sciences, University of Alabama at Birmingham, Birmingham, AL 35294, USA; jkwickli@uab.edu; 9 Department of Laboratory Medicine and Pathology, University of Minnesota, Minneapolis, MN 55455, USA

**Keywords:** lead isotopes, dried blood spots, biomarkers, blood collection, Suriname, child health

## Abstract

**Background**: Lead (Pb) exposure remains a major global health issue, and Pb stable isotope ratio analysis is a powerful tool for identifying potential exposure sources. However, traditional whole blood collection is difficult to implement in remote or resource-limited settings. We developed and validated a novel method for measuring Pb isotopic composition in dried blood spots (DBS) using a multi-collector inductively coupled plasma mass spectrometer (MC-ICP-MS) and applied it in a pilot study of DBS collected from children in Suriname, South America. **Methods**: Method accuracy was evaluated by comparing isotopic ratios (^208^Pb/^206^Pb and ^207^Pb/^206^Pb) measured in laboratory-spiked DBS (15 µL of blood per DBS) with those from matched whole blood samples (1 mL) across a range of Pb concentrations (2.1–28.9 µg/dL). The method was then applied to DBS collected from 18 children, ages 6 months to 5 years, in Suriname. Potential local exposure sources, including soil, shotgun pellets, and cooking utensils, were also analyzed for lead isotopic composition and compared with DBS isotope ratios to assess source concordance. **Results**: DBS isotopic ratios accurately reflected whole blood values when Pb concentrations exceeded ~315 pg/punch (~2.1 µg/dL), with greater accuracy observed above 5 µg/dL. In the Suriname DBSs, blank-corrected ^208^Pb/^206^Pb ratios were consistent across regions (mean 2.12 ± 0.018; n = 15) and showed no significant geographic stratification (*p* = 0.11). DBS signatures (isotope ratios) most closely aligned with shotgun pellets and soil samples, whereas those from cooking utensils did not match DBS signatures. A positive correlation was observed between estimated blood total Pb and soil Pb concentrations (ρ = 0.60, *p* = 0.20). **Conclusions**: Overall, DBS provided reliable Pb isotopic ratio measurements above ~2–5 µg/dL, spanning the current CDC reference value of 3.5 µg/dL at which children’s exposures warrant investigation. These findings support DBS as a minimally invasive, scalable tool for exposure source identification in environmental epidemiology, particularly in remote settings where traditional venipuncture is impractical. **What this study adds**: This study establishes the first validated method for high-precision lead (Pb) stable isotopic ratio analysis using dried blood spots (DBS). By implementing a sample blank correction, we demonstrate that DBS can accurately resolve isotopic signatures even at low blood lead concentrations. Applied in DBS collected in Suriname, the method successfully linked DBS signatures to local shotgun pellets and soils, illustrating a common exposure profile across diverse regions. This work expands the utility of DBS beyond simple screening, offering a minimally invasive, scalable tool for source attribution in global health research and environmental epidemiology in remote regions.

## 1. Introduction

Human exposure to lead (Pb) remains a persistent public health concern that disproportionately affects populations in transition [1]. Although environmental regulations have reduced Pb exposure in many countries, Pb remains widespread through contaminated drinking water, soil, household dust, legacy lead-based paint, and ongoing industrial or occupational sources [2,3,4,5]. In many low- and middle-income countries (LMICs), the lack of robust environmental regulations and surveillance means that vulnerable populations often bear a disproportionate share of the global Pb burden [6,7]. Identifying and eliminating exposure to Pb in these regions is often hindered by a lack of diagnostic tools capable of distinguishing between multiple potential sources. Childhood Pb exposure is known to result in significant long-term neurodevelopmental and socioeconomic consequences; however, effective mitigation is only possible when the specific environmental drivers are accurately identified [8]. Consequently, there is a need for accessible, high-precision methods that move beyond simple screening for elevated blood Pb to trace Pb exposure back to its environmental sources.

Pb exposure is associated with numerous adverse health outcomes. In children, even low-level exposure causes lifelong neurodevelopmental impairments, including reduced IQ and attention deficits, effects that persist into adulthood when exposure occurs during critical developmental windows [9,10]. Pb exposure is also linked to reproductive harms, including infertility and miscarriage [11,12]. Our previous work in the Republic of Suriname identified a significant geographic disparity in Pb exposure. Pregnant women living in the Amazonian Interior had significantly higher median blood lead levels (5.08 µg/dL) as compared to those in urban (1.77 µg/dL) and suburban (1.79 µg/dL) regions [13,14]. This disparity highlights an urgent environmental health need to identify local sources of Pb exposure to inform mitigation strategies. Suspected sources of Pb exposure in Amazonian communities include manioc-derived foods, wild game (harvested with Pb-shot), metal cooking utensils, and the traditional consumption of kaolin clay (locally known as pimba) [15,16,17,18]. Given the diversity of potential exposure sources, robust methods for source identification or “fingerprinting” are essential to prioritize which environmental interventions will most effectively reduce pediatric lead burdens.

Naturally occurring Pb consists of four stable isotopes (^204^Pb, ^206^Pb, ^207^Pb, and ^208^Pb) whose relative abundances vary with ore geology and smelting history, producing source-specific isotopic “signatures”. Fractionation during environmental and biological processes is minimal for Pb; therefore, isotopic ratios in environmental samples (e.g., soil, water, ammunition, etc.) are generally preserved in human tissues. High-precision Pb isotopic ratio analysis is a well-established “fingerprinting” approach for identifying sources of lead exposure. Studies, including our child risk investigations, have successfully traced the origins of Pb exposure by comparing the isotopic signatures (^208^Pb/^206^Pb and ^207^Pb/^206^Pb) in human blood to potential environmental sources including lead-based paint, soils, gasoline, drinking water, cosmetics and traditional remedies, ceramics, bullet fragments, and diet [16,19,20,21,22,23,24,25,26,27,28,29,30,31]. Traditionally, this approach requires venous whole blood collection, which can be difficult to implement in resource-limited or remote settings where trained phlebotomists, clean and sterile collection materials, and uninterrupted cold chain storage may be unavailable. Dried blood spots (DBS), collected via a finger or heel prick, offer a practical, minimally invasive alternative. By enabling field-friendly collection of specimens suitable for isotopic analysis, DBS creates an opportunity to extend source-attribution approaches to settings where only basic screening has previously been feasible. Despite their increasing use in exposure assessment, the utility of DBSs for Pb isotopic fingerprinting has not been evaluated in real-world field settings [32].

In this study, we developed and validated a method for measuring Pb isotopic composition in DBS as an alternative to whole blood using multi-collector inductively coupled plasma mass spectrometry (MC-ICP-MS). We demonstrate the accuracy of this approach through laboratory validation and apply it to DBS collected from a pilot cohort of children in Suriname. Our primary objective was to demonstrate that DBS-based isotopic analysis can be used to identify exposure sources particularly in remote populations, transforming a logistical challenge into a public health opportunity.

## 2. Materials and Methods

### 2.1. Method Development and Validation

#### 2.1.1. Dried Blood Spot Preparation

All procedures were carried out in the Trace Elements Clean Lab (TECL) at the Wisconsin State Laboratory of Hygiene (WSLH), a facility dedicated to ultra-low-level elemental and stable isotopic analysis of inorganic constituents (see Supplementary Materials). To validate methods, we simulated a physiological relevant range of pediatric exposures, by using bovine whole blood from the WSLH National Blood Pb Proficiency Testing Program [33] at four Pb concentrations: 2.1, 5.7, 14.4, and 28.9 µg/dL (Table S1). These concentrations span below and above the current U.S. Center for Disease Control (CDC) blood Pb level of concern of 3.5 µg/dL [34].

Precisely, 50 µL aliquots of bovine whole blood were pipetted onto Whatman^TM^ 903 Protein Saver filter paper cards (Lot 7177620 W191; Figure S1). Cards were dried overnight in a HEPA-filtered laminar flow hood. Each DBS was sampled using an 8 mm stainless steel Rapid-Core punch (Electron Microscopy Sciences, Hatfield, PA, USA), corresponding to approximately 15 µL of blood per punch. Blank card punches were collected from adjacent un-spotted card areas to quantify background Pb (Figure S1). For accuracy and reproducibility, we analyzed NIST SRM 981 (National Institute of Standards and Technology, Common Lead Isotopic Standard, Gaithersburg, MD, USA) [35] and two UTAK whole blood Pb reference materials (2 µg/dL [Lot A9198] and 41 µg/dL [Lot 9852]; UTAK Laboratories, Valencia, CA, USA). We also analyzed DLS-1 (Devil’s Lake Soil), an internal geologic reference material collected from Devil’s Lake, Wisconsin.

#### 2.1.2. Sample Digestion and Pb Purification

Each DBS punch and corresponding blank punch were digested in 2 milliliters (mL) of 16 M ultra-trace-grade nitric acid (HNO_3_; Optima^TM^ Fisher Chemical, Thermo Fisher Scientific, Fair Lawn, NJ, USA). Unspotted reference whole blood aliquots (0.7 mL) were digested in 3 mL of HNO_3_. Samples were placed in Teflon jars, acid was added, and they were then pre-digested overnight at room temperature and heated to 110 °C for a second night.

Lead was isolated using anion-exchange chromatography following a modified version of established protocols [24]. Briefly, digested samples were evaporated and reconstituted in 0.6 mL of 1 M hydrobromic acid (HBr; Optima^TM^ Fisher Chemical, Thermo Fisher Scientific, Fair Lawn, NJ, USA) before loading onto Bio-Rad Poly-prep^®^ columns containing 0.6 mL AG1-X8 resin (Bio-Rad Laboratories, Hercules, CA, USA). Matrix elements were removed with successive washes of 1 M HBr (0.6 mL, 1.2 mL, 1.2 mL, and 4.8 mL). Pb was eluted using two 0.6 mL washes of deionized water followed by two 3.6 mL washes of 1 M HNO_3_. Purified fractions were evaporated and reconstituted in 2 mL of 2% HNO_3_ for analysis.

#### 2.1.3. Quantification of Pb Isotopic Ratios

High-precision stable isotope ratio analysis was performed on a Neptune Plus MC-ICP-MS (Thermo Fisher Scientific Inc., Waltham, MA, USA) using well-established methods [25,36,37]. These MC-ICP-MS protocols closely aligned with those previously validated by our team for Pb isotope analysis in whole blood, animal tissues, and environmental matrices, where high analytical precision and robustness have been demonstrated [30,38,39]. To quantify the reliability of DBS as a proxy for whole blood Pb isotope measures, we calculated Pb isotopic deviation (ΔPb) between paired spotted (S) and reference whole blood (WB) samples using the following equation:

ΔPb=Pb207Pb206S−Pb207Pb206WB2+Pb208Pb206S−Pb208Pb206WB2

In this expression, Pb207Pb206S and Pb208Pb206S are the isotope ratios measured in the DBS (spotted) sample, and Pb207Pb206WB and Pb208Pb206WB are the corresponding ratios measured in the matched reference whole blood sample. The metric is calculated as the square root of the sum of squared differences between the DBS and whole blood ratios and therefore represents the Euclidean distance between the two samples in two-dimensional Pb-isotope-ratio space. Analytical uncertainties for individual isotopic ratios ranged from 0.002% to 0.017% (2σ, two-sigma level; Table S1). Based on background-corrected beam intensities, the instrumental detection limit was approximately 2.5 pg Pb per mL of final solution. Assuming a nominal DBS volume of 15 µL of blood per 8 mm punch and a final digest volume of 1 mL, this corresponds to an equivalent blood Pb detection limit of about 0.02 µg/dL, which is more than two orders of magnitude lower than our lowest validation spike (2.1 µg/dL). Detailed instrument configurations and mass bias correction protocol using a Thallium (Tl) spike (^205^Tl/^203^Tl = 2.387) are provided in Supplementary Materials.

### 2.2. Application of Lead Isotope Analysis to the Dried Blood Spots (DBS) from Suriname Children

#### 2.2.1. Participant Selection and Field Collection

This pilot study utilized DBS and environmental samples from the Caribbean Consortium for Research in Environmental and Occupational Health (CCREOH) cohort [14]. Samples for this sub-study were collected from 18 children (ages 6 months to 5 years) from an existing cohort of pregnant women living in Suriname with previously measured whole blood Pb levels ranging from 5 to 34 µg/dL [13]. These participants were stratified into two geographic groups: Interior children (n = 6), residing in Amazonian Interior communities where higher Pb exposure was previously identified, and Non-Amazonian Interior children (n = 12), representing more urban or coastal regions. Selection criteria incorporated ethnicity (Tribal and Indigenous), geographic residence, and representation across both high and low Pb exposure (as evidenced by whole blood lead levels [13]). Six of the Interior children also had paired environmental samples (soil, pellets, and utensils) collected from their homes to facilitate regional source attribution. No additional questionnaire data were collected for this study. Written informed consent was obtained from the mother or primary caretaker of each child for the overall CCREOH cohort study (Study ID: VG023-14).

DBS samples were collected by trained CCREOH study staff between January and June 2021 via a finger prick following a standardized field protocol (see Supplementary Materials “Blood Collection and Handling—Dried Blood Spot”). Samples were initially held under climate-controlled conditions, then transferred to −80 °C storage in Paramaribo at the Clinical Chemistry Laboratory of the Academic Hospital and shipped to the WSLH via a formal chain-of-custody process.

#### 2.2.2. Environmental Source Sampling

To facilitate source attribution, environmental samples were collected from the residential areas of six children:

(1) Cooking utensils: Cassava grater and pans.
(2) Shotgun pellets: Ammunition used for hunting game were obtained from local sources; study staff removed pellets from cartridges or collected spent material for analysis.
(3) Surface soil: Samples were collected from the residential yards and children’s play areas around the homes of the children providing DBS samples. Soil was stored in pre-cleaned polyethylene bags prior to shipment.

#### 2.2.3. Laboratory Processing of Suriname Samples

Environmental and biological samples were processed in parallel with bovine QC materials following the methods described above (see “Method Development: Section 2.1.2 Sample digestion and Pb purification and Section 2.1.3 Quantification of Pb isotopic ratios”).

Shotgun pellets and cooking utensils were extracted using a well-established, non-destructive method commonly used for archaeological artifacts [40]. Briefly, metal samples were subsampled using wire cutting tools, cleaned in purified water, weighed into pre-cleaned PFA jars, and extracted in 5 mL ethylenediaminetetraacetic acid (EDTA; Sigma-Aldrich, St. Louis, MO USA) solution overnight. NIST SRM 981 wire was processed alongside as a control.

Soil Samples (0.1–2 g) were digested in 2 mL hydrofluoric acid (HF; Optima^TM^ Fisher Chemical, Thermo Fisher Scientific, Fair Lawn, NJ, USA) and 5 mL HNO_3_ at 110 °C, following a modified protocol based on our previous analyses [30,39]. Digests were then redissolved in 5 mL of 6 M hydrochloric acid (HCl; Optima^TM^ Fisher Chemical, Thermo Fisher Scientific, Fair Lawn, NJ, USA). Each soil digest was split: one-tenth of the digest was spiked with ^206^Pb for concentration measurement via isotope dilution, while nine-tenths were used for isotopic composition. All digests for isotopic characterization were purified using the anion-exchange chromatography described above (see “Section 2.1.2 Sample digestion and Isotopic analysis”).

## 3. Results

### 3.1. Method Development: Pb Isotopic Accuracy in DBS

**Figure 1:**
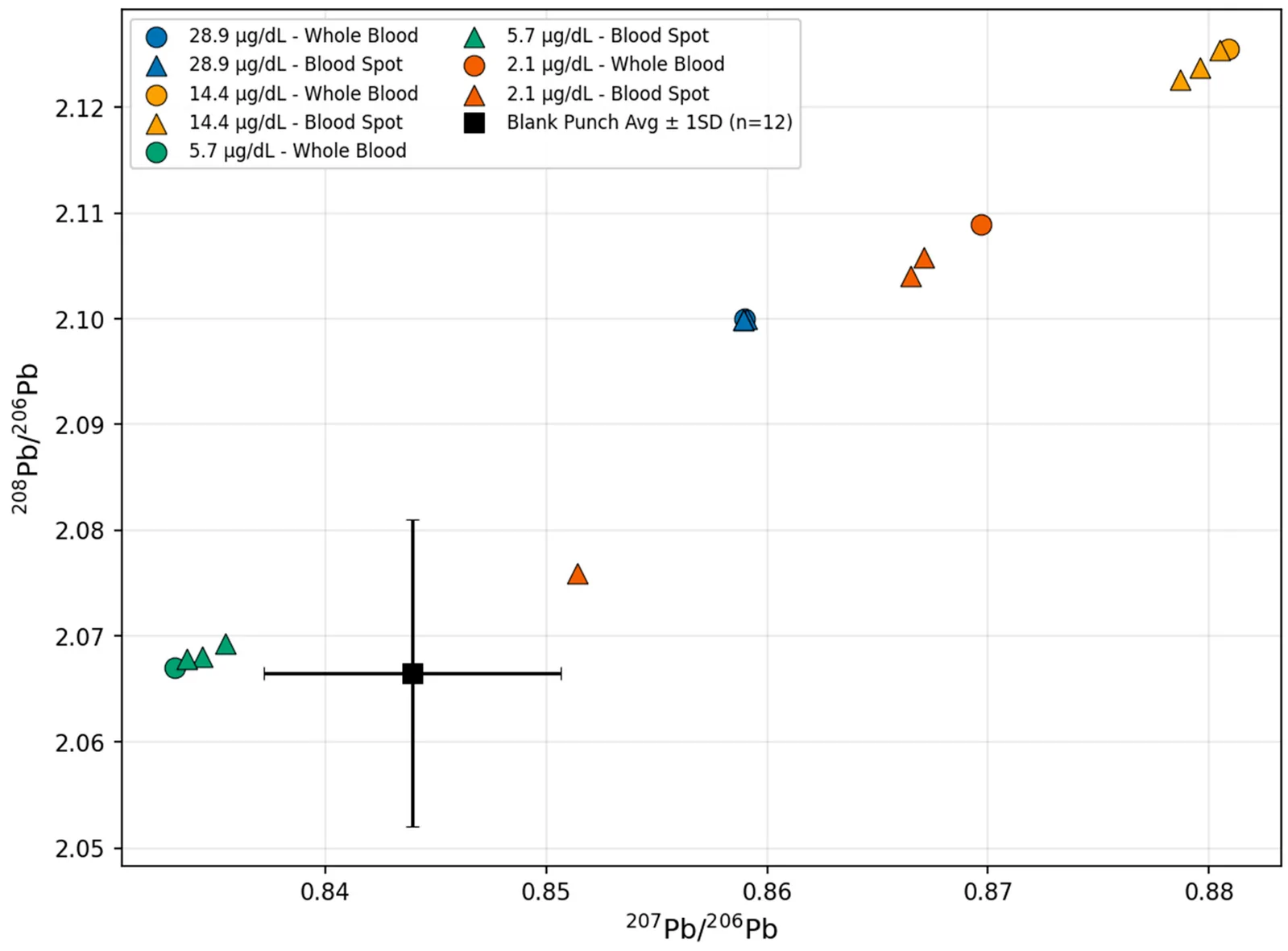
**Method development samples.** Plot of ^208^Pb/^206^Pb vs. ^207^Pb/^206^Pb for whole blood, blood spots, and average blank punches. At 28.9 µg/dL, whole blood and DBS compositions overlap completely. At 2.1 µg/dL, DBS samples show deviation from whole blood due to blank contributions. Analytical uncertainties of individual data points are smaller than the symbol.

The stable Pb isotopic compositions of laboratory-controlled reference whole blood (standard reference material [SRM] and bovine blood) samples were distinct from one another and remained consistent when spotted on filter paper (Figure 1 and Figure S2 and Table S1). We observed that the DBS Pb isotopic composition accuracy was significantly influenced by the total Pb content within the 8 mm punch. Specifically, the isotopic deviation (∆Pb) between DBS and bovine whole blood increased and became more variable as blood Pb concentrations decreased (Figure S2). At the lowest bovine whole blood Pb level (2.1 µg/dL, representing ~315 pg total Pb per 8 mm punch), ∆Pb ranged from 0.4% to 3.8% (Figure 1, Table S1). The Pb isotopic composition of blank card punches varied, but this variation did not show a systematic pattern across different blank card punches. However, blank variability was substantial in comparison to low-concentration DBS samples and therefore precluded accurate blank correction for these low-concentration DBS samples (Table S1). The ^208^Pb/^206^Pb ratios of all blank punches ranged from 2.046 to 2.094 (mean 2.067 ± 0.029; 2 standard deviations [SD], n = 12), whereas the ^207^Pb/^206^Pb ratio ranged from 0.835 to 0.851 (mean 0.844 ± 0.013, 2SD, n = 12; Table S1).

To confirm that relatively higher ∆Pb values at low concentrations were not due to instrumental error, we analyzed NIST SRM 981 standard reference solutions at concentrations below those found in the DBS samples. These analyses never exceeded a ∆Pb of 0.0033 (Figure S3), confirming that the observed deviations in DBS samples were attributable to filter paper background rather than analytical uncertainty. Furthermore, analyses of UTAK reference whole blood materials (2 µg/dL and 41 µg/dL) also yielded highly reproducible values for all Pb isotope ratios, with ∆Pb ranging from only 0.0002 to 0.0006 (Table S2).

### 3.2. Application: Pb Isotopic Composition in Suriname DBS Samples

We applied this method to DBS samples collected from 18 children in Suriname to evaluate method robustness in a field setting.

#### 3.2.1. DBS vs. Adjacent Blanks

**Figure 2:**
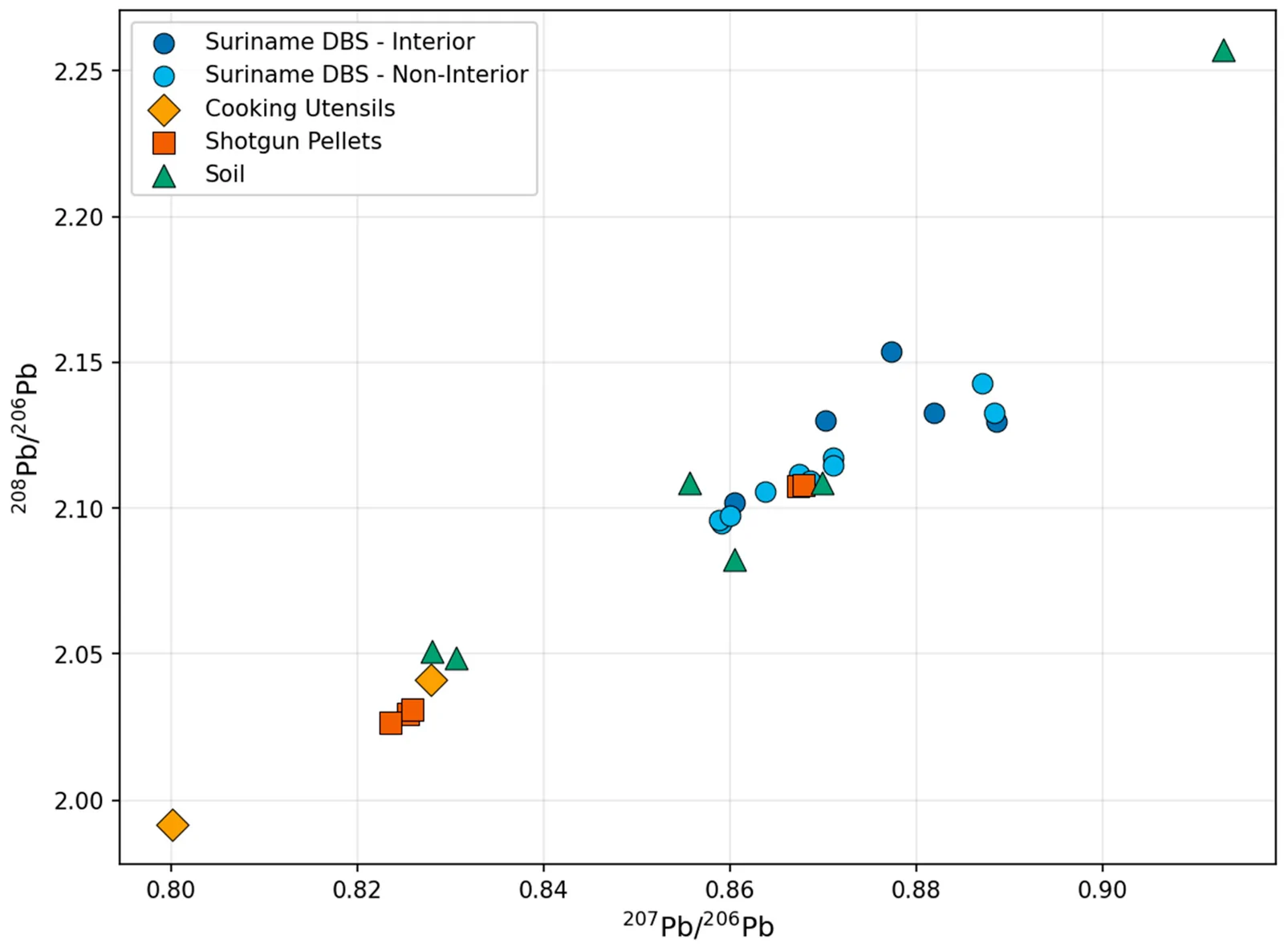
**Lead (Pb) Isotopic signatures of DBS from children and potential environmental sources in Suriname.** Plots of ^208^Pb/^206^Pb versus ^207^Pb/^206^Pb showing blank-corrected DBS samples (n = 15) and potential environmental sources (n = 13). Samples from children residing in the Amazonian Interior (n = 5) are individually labeled to facilitate direct comparison with household-level environmental signatures (e.g., cooking utensils, soil and shotgun pellets).

The isotopic compositions of the Surinamese DBS samples were generally distinct from their adjacent blank punches, characterized by less radiogenic ratios (Figure 2). Raw (not blank-corrected) ^208^Pb/^206^Pb ratios for DBS ranged from 2.06 to 2.15, whereas adjacent blanks ranged from 1.92 to 2.12. Field-collected blank card punches showed considerably more variation than blank punches from the laboratory-controlled setting (Table S3). The influence of the filter paper matrix on the raw isotopic signal is shown in Figure S4. The shift from uncorrected (grey) to blank-corrected (red) values illustrates the removal of the Pb contribution from the filter paper. Note, samples were blank-corrected using its corresponding card blank and an atom-basis subtraction to account for isotope-specific background interference.

Despite this background variability, the blank-corrected ^208^Pb/^206^Pb ratios for the DBS samples from the cohort remained consistent, ranging from 2.09 to 2.15 (mean 2.12 ± 0.018, 1 SD, n = 15; Table S3). Stratification by geography showed that mean ratios for children in the Amazonian Interior (2.13 ± 0.02, n = 5) were isotopically indistinguishable from those in non-Interior regions (2.11 ± 0.02, n = 10; *t*-test *p* = 0.11), suggesting exposure sources with similar geochemical signatures. The isotopic composition difference between each Surinamese DBS and its corresponding blank punch ranged from 0.6% to 9.3%, which is substantial relative to MC-ICP-MS analytical uncertainties. In three DBS samples, the blank card Pb signal (range 0.32–1.87 ng total Pb) exceeded the corresponding DBS signal (range 0.31–2.36 ng total Pb), rendering blank-corrected values unreliable; therefore, these samples are not shown in Figure 2 or Figure S4 (data in Table S4). Estimated total blood Pb levels for the corrected DBS (assuming 15 µL of bovine whole blood equivalent) ranged from 1.6 to 33.4 µg/dL. The total Pb in adjacent blanks in the DBS ranged from 2 to 195% of the total DBS Pb (median 14%).

#### 3.2.2. Environmental Source Characterization

Environmental samples collected from the Interior communities demonstrated a broad isotopic composition range (Figure 2 and Table S4) with ^208^Pb/^206^Pb ratios spanning 1.98 (cassava grater) to 2.25 (soil). Notably, two shotgun pellet samples (^208^Pb/^206^Pb ~ 2.11) and two soil samples (^208^Pb/^206^Pb ~ 2.11) yielded isotopic signatures most similar to the signatures observed in the Suriname DBS samples (Figure 2). While environmental source attribution was focused on the Interior, the isotopic homogeneity observed across the entire Surinamese cohort suggests that potentially modifiable household and environmental sources may contribute to child Pb exposure in Suriname. Total Pb concentration was not measured for pellets or cooking utensils because the non-destructive nature of the analysis; however, Pb concentrations in soil samples (determined by isotope dilution) ranged from 1.2 to 27.3 µg/g (median 14.6 µg/g). These levels are below the U.S. Environmental Protection Agency (EPA) residential soil hazard threshold of 200 µg/g [41]. Preliminary correlation analysis of the limited paired samples suggested a moderately strong positive relationship between total Pb concentration in DBS and soil, though it did not reach statistical significance (ρ = 0.60, *p* = 0.204).

## 4. Discussion

In this study, we developed and validated a novel method for determining stable Pb isotopic composition in DBS as an alternative to whole blood using MC-ICP-MS. Our method allows for the precise and accurate quantification of stable Pb isotope ratios across a wide range of Pb concentrations, including levels below the current CDC blood Pb reference level of 3.5 µg/dL (noting that no safe level of Pb exposure in children has been established) [34]. The method is not intended for precise quantification of blood Pb concentrations, though it can still provide actionable isotopic data at low concentrations relevant for exposure assessment. As such, DBS isotope measurements are best interpreted as a research and source-attribution tool that complements, rather than replaces, validated clinical assays for blood Pb concentration. Pb isotopic composition in DBS demonstrated strong concordance with matched reference whole blood samples, particularly above ~5 µg/dL. Our validation focused on analytical accuracy of Pb isotopic ratios through spike-recovery experiments, comparison with matched whole blood samples, and characterization of blank contributions. Formal intra-day and inter-day precision experiments were not performed in this pilot; instead, we relied on previously validated MC-ICP-MS Pb isotope protocols we developed for blood and environmental matrices, and a full assessment of assay precision and robustness will be an important focus of future method-development work. Furthermore, in the Suriname pilot, this approach enabled identification of potential Pb exposure sources by comparing isotopic signatures in DBS with those of environmental samples. Taken together with our previous applications of MC-ICP-MS Pb isotope analysis in individual child risk assessments [30], these findings support the feasibility of using DBS as a minimally invasive, field-adaptable matrix for Pb source attribution, and suggest that isotopic fingerprinting can complement conventional investigations by helping to identify plausible dominant sources in complex exposure settings. Unlike previous Pb isotope investigations that relied on venous whole blood, this study demonstrates the feasibility of using DBS as a field-collected matrix for isotopic source attribution in young children.

Whole blood isotope ratios are well established as indicators of external Pb sources [16,19,20,21,22,23,26,27,28,29], and our validation demonstrates that DBSs are a viable matrix for this analysis. This extends isotope-based source attribution to contexts where venous blood collection is impractical, especially important in low-resource and geographically isolated settings where conventional whole-blood collection and storage require trained phlebotomist, clean and sterile supplies, and cold-chain infrastructure. In contrast, DBS samples can be collected with minimal equipment and stored at ambient temperature.

An important observation was the elevated total Pb values at the lowest spiked Pb concentrations (<2.1 µg/dL), which we attributed to Pb present in the filter card blanks, consistent with the prior literature on background contamination in DBS material [42,43]. This finding highlights that filter paper contamination imposes a practical lower limit of quantification for both Pb concentrations and isotopic ratios in DBS, despite the high precision of the MC-ICP-MS measurements. These calculations indicate that the instrumental detection limit (~0.02 µg/dL in blood equivalents) is far below the lowest validation spike (2.1 µg/dL), highlighting that filter paper and other background contributions, rather than MC-ICP-MS sensitivity, are the primary constraints on DBS performance at very low Pb levels. Thus, this underscores a critical distinction: while the DBS assay is highly robust for characterizing isotopic signatures of Pb exposure (i.e., identifying which sources contribute) at moderate and higher concentrations, its analytical sensitivity at very low Pb levels is constrained by inherent background Pb levels in the card material and/or potentially from external contamination. Future work should incorporate a gradient blank design, including unused-card, field-transport, and digestion blanks, to refine blank correction and improve the design of low-concentration quality control experiments. In practice, this suggests that DBS-based isotopic analysis is the most informative when used alongside established low-level Pb quantification assays, rather than as a stand-alone tool for defining individual exposure limits, particularly below the range where blank correction remains reliable.

In the children from the Surinamese Interior, Pb isotope composition in DBS more closely resembled signatures found in soil and shotgun pellets. This finding was supported by the subset correlation analysis of paired samples, which suggested a moderately strong, though non-significant, positive association between estimated total Pb concentration in DBS and soil. Taken together, these isotopic clustering and correlation results suggest that soil is an important exposure source in this population, potentially through ingestion of contaminated soil or household dust of soil origin, recognizing that soil Pb was quantified as total Pb rather than bioaccessible fractions and thus reflects contamination levels rather than directly measured absorbed dose from soil ingestion. In addition, multiple pathways associated with hunting and shooting environments, including consumption of pellet-contaminated game and dermal-to-mouth transfer, likely contribute to Pb exposure. These patterns align with a “multi-source” exposure model common in communities reliant on subsistence activities, where geogenic dust and ammunition-related Pb exposures may co-occur [31,44]. However, because only a limited set of environmental samples was sampled in this pilot, the isotopic signatures observed here should be interpreted as suggestive rather than definitive evidence of specific source contribution. Three child DBS samples with blank-corrected Pb signals indistinguishable from or lower than the corresponding card blanks were therefore excluded from isotopic analysis, reducing the effective sample size for field comparisons but ensuring that only measurements exceeding total blank contributions were interpreted.

The Amazonian Interior is characterized by artisanal and small-scale gold mining and other activities that may contribute to metal contamination in locally imported foods such as fish and manioc. Previous work in Suriname and neighboring French Guiana has documented elevated blood levels of both Pb (often alongside Hg) among pregnant women, with suggestive exposure pathways including consumption of manioc (also known as cassava), wild game, and kaolin clay (pimba) [45,46]. These findings demonstrate the utility of isotopic profiling for identifying plausible exposure sources in vulnerable populations. However, overlapping isotopic signatures among environmental sources and the likelihood of mixed-source exposure means that these results should be interpreted as identifying the most plausible dominant sources rather than providing definitive, single-source attribution [47,48]. Despite these environmental concerns, regulations on toxic substances remain limited, and comprehensive environmental health data are sparse, particularly for Indigenous and remote communities. Although we did not collect fish, manioc-derived products, wild game, or pimba in this pilot, potential dietary contributions to Pb exposure could not be directly evaluated, and our results instead highlight these pathways as priority targets for future community-based research.

Several limitations should be acknowledged. Environmental sampling was constrained, and other potential sources such as drinking water, game meat, cassava-derived products, and household dust were not collected, limiting the ability to fully characterize exposure pathways. In addition, soil Pb measurements reflected total Pb concentrations rather than bioaccessible fractions, precluding direct estimation of the fraction of ingested soil Pb that would be solubilized in the gastrointestinal tract and contribute to absorbed dose among children. Together with the limited number of environmental samples analyzed, these factors introduce uncertainty into source-attribution inferences based on isotopic clustering. Overlapping isotopic signatures among environmental sources and the likelihood of mixed-source exposures present inherent challenges of Pb isotope-based source attribution. The one-time collection of DBS samples also restricts assessment of temporal variation in Pb exposure. In addition, as a pilot study with a relatively small sample size, statistical power for correlation analyses was limited, which may affect the generalizability of the findings. Despite these limitations, this study represents one of the first applications of Pb isotope ratio analysis to DBS in a pediatric population in Suriname and illustrates a practical framework for integrating biological and environmental data to identify potential Pb exposure sources. The use of established mass spectrometry techniques, paired DBS–whole blood samples, and environmental measurements provides a strong analytical foundation for future, larger-scale studies.

In conclusion, this study provides a practical analytical method for extending Pb isotope biomonitoring to settings where conventional venipuncture-based methods are impractical. With careful attention to quality control, particularly characterization and correction of card blank Pb, DBS-based isotope analysis offers a powerful tool for both clinical follow-up of individuals with elevated Pb and identification of likely environmental sources of Pb exposure in underserved populations, including Indigenous and remote communities. By integrating DBS sampling with high-precision MC-ICP-MS, this approach has the potential to shift the clinical utility of DBS from simple “high/”low” screening toward a tool capable of identifying environmental drivers of toxicity and informing targeted remediation [42,49,50,51]. In practice, isotopic ratio data can help identify likely exposure sources and prioritize public health actions. Beyond the Suriname setting, this approach could be applied to other populations where venous sampling is logistically challenging, including large-scale surveillance programs that already rely on DBS for newborn or pediatric screening. Future studies incorporating larger sample sizes, repeated sampling, and more extensive environmental and dietary measurements, along with more detailed blank experiments, will refine Pb source models and support targeted policy and community-level actions to reduce Pb exposure.

## Data Availability

Data are available from the corresponding author on reasonable request. CCREOH Cohort study data will be made publicly available upon publication of the results in scientific articles. Questionnaire, registry and biospecimen data could be made accessible in deidentified form after an application process that includes submission of a research plan. We are currently collaborating with Research Triangle Institute International, a global data management enterprise, to develop an integrated database of biospecimen and non-biospecimen data. Once that is fully developed, data can be made available based on a reasonable request to the Principal Investigators (Drs. Maureen Lichtveld, mlichtve@pitt.edu and Wilco Zijlmans, wilco.zijlmans@uvs.edu). Such requests will be discussed with the full investigator Committee, the Data Management Committee and the Administrative Oversight Committee.

## Supplementary Materials

The following supporting information can be downloaded at: https://www.mdpi.com/article/10.3390/toxics14080715/s1, Figure S1: Whatman^TM^ filter paper card commonly used for newborn screening, shown after being spotted with blood and has several 8 mm punches removed for analysis; Figure S2: Method Development Samples. Comparison of isotopic deviation (∆Pb) versus blood Pb concentration (µg/dL) for spotted blood samples. ∆Pb increases as blood Pb concentrations decrease, reflecting greater and more variable blank contributions from the filter paper. Analytical uncertainties of individual data points are smaller than the symbol sizes; Figure S3: Analytical Validation. Comparison of isotopic deviation (∆Pb) values vs. Pb concentration in NIST SRM 981 standard reference material solutions. The low ∆Pb (<0.004) across all concentrations confirms that DBS deviations are environmental/matrix-based rather than analytical error; Figure S4: Suriname Data. Plot showing ^208^Pb/^206^Pb vs. ^207^Pb/^206^Pb in Suriname dried blood spots before (grey) and after (red) an atom-basis blank correction for filter paper contributions. Each DBS was corrected using its specific adjacent card blank to account for localized filter paper Pb contributions. Faint tie-line (blank-correction vectors) connect paired data points, illustrating the magnitude and direction of the isotopic shift resulting from the recalculation. The consistent upward and rightward trajectory of the vectors confirms that the filter paper background was systematically less radiogenic than the DBS Pb. Vector length reflects the relative influence of the blank atoms on the Pb isotopic composition of the sample. Table S1: Lead (Pb) isotope results for method development samples using reference material including whole blood, blood spots, and blank card punches; Table S2: Lead (Pb) isotopic analysis of whole blood reference materials; Table S3: Summary Demographic data for Suriname children samples (N = 18); Table S4: Lead (Pb) Dried Blood Spot Data from Suriname Children Dried Blood Spots; Table S5: Environmental sample collection from the Amazonian Interior, Suriname. Reference [52] is cited in the Supplementary Materials.
