# Supplementary material for: Advancing Lead Exposure Studies in Remote Settings: Method Development and Application of Lead Stable Isotope Analysis in Dried Blood Spots from Suriname, South America
Source: Toxics. 2026 Aug 13;14(8):715. doi: 10.3390/toxics14080715 (PMC13517531; doi:10.3390/toxics14080715)
Supplement: Supplementary file 1 [file toxics-14-00715-s001.zip › toxics-4443134-supplementary_figures.pdf]

Article

Advancing Lead Exposure Studies in Remote Settings: Method Development and Application of Lead Stable Isotope Analysis in Dried Blood Spots from Suriname, South America

Manasi Simhan [1,2], Sean R. Scott [2], Martin M. Shafer [2], Jenny N. Poynter [3], Gaitree K. Baldewsingh [4], Wilco C. W. R. Zijlmans [5,6], Anisma R. Gokoel [6], Maureen Y. Lichtveld [7], Jeffrey K. Wickliffe [8] and Shannon M. Sullivan [3,9,*]

1 Nelson Institute for Environmental Studies, University of Wisconsin-Madison, Madison, WI 53707, USA

2 Wisconsin State Laboratory of Hygiene, School of Medicine & Public Health, University of
Wisconsin-Madison, Madison, WI 53718, USA; mmshafer@wisc.edu (M.M.S.)

3 Department of Pediatrics, University of Minnesota, Minneapolis, MN 55455, USA; poynter@umn.edu

4 Medical Mission Primary Health Care Suriname, Paramaribo, Suriname; gbaldewsingh@medischezending.sr

5 Foundation for Perinatal Interventions and Research in Suriname, Paramaribo, Suriname;
wilco.zijlmans@uvs.edu

6 Faculty of Medical Sciences, Anton de Kom University of Suriname, Paramaribo, Suriname;
anishmag@gmail.com

7 School of Public Health, University of Pittsburgh, Pittsburgh, PA 15267, USA; mlichtve@pitt.edu

8 School of Public Health, Department of Environmental Health Sciences, University of Alabama at
Birmingham, Birmingham, AL 35294, USA; jkwickli@uab.edu

9 Department of Laboratory Medicine and Pathology, University of Minnesota, Minneapolis, MN 55455, USA

* Correspondence: sull0401@umn.edu; Tel.: +1-612-626-0320

Supplementary Methods

Laboratory Environment and Cleaning Procedures

All sample handling and preparation were conducted in the TECL at WSLH. Sample preparation, digestion, and ion-exchange procedures occurred in a class-1000 ultra-clean room, while isotope measurements were performed in a class-10,000 instrument room. Airborne particle counts were routinely monitored to ensure cleanroom standards.

Detailed MC-ICP-MS Analytical Protocol

Pb isotopic analyses were performed on a Neptune Plus MC-ICP-MS (Thermo Fisher Scientific Inc., Waltham, MA) equipped with an Aridus 3 desolvating nebulizer system (Teledyne Cetac), Jet sampler cone, and X-series skimmer cone. This configuration typically yields sensitivities of ~3000- 4000 V/parts per million (ppm) total Pb. A Thallium (Tl) spike was added to each sample to achieve a Pb/Tl ratio of approximately 3 to correct for instrumental mass bias using the exponential fractionation law, assuming $^{205}Tl/^{203}Tl = 2.387$. Pb and Tl isotopes were analyzed in static mode using six Faraday collectors, with a seventh collector used to monitor Mercury (Hg) interferences. Beam intensities were measured for 60 cycles with 8-second integration times per cycle. NIST SRM 981 (Common Lead Isotopic Standard; National Institute of Standards and Technology) [35] was analyzed at the start and periodically throughout each analytical run. Results were reported relative to the SRM 981 ratios established by Thirlwall [52]. The method limit of detection (LOD) for blanks was estimated at ~5 picograms (pg) of total Pb.

Pb isotope accuracy at low concentrations

To access accuracy at low Pb concentrations, NIST SRM 981 was prepared at concentrations of 2.5, 1.0, 0.5, 0.25, 0.15, and 0.05 ng/g Pb and measured in triplicate. Deviations between low concentrations of NIST SRM 981 (*X*) were calculated relative to a 5 ng/g reference solution (*5*) using the following equation:

$$\Delta Pb = \sqrt{\left(\left[\frac{^{207}Pb}{^{206}Pb}\right]_X - \left[\frac{^{207}Pb}{^{206}Pb}\right]_5\right)^2 + \left(\left[\frac{^{208}Pb}{^{206}Pb}\right]_X - \left[\frac{^{208}Pb}{^{206}Pb}\right]_5\right)^2}$$

The maximum deviation (ΔPb = 0.0033) at 0.05 ng/g was still lower than the ΔPb range observed in the lowest blood Pb samples (0.0040 to 0.0377; **Supplementary Figure S4**), confirming that observed deviations stem from filter paper background rather than analytical uncertainty. These data show that the $^{208}Pb/^{206}Pb$ and $^{207}Pb/^{206}Pb$ values can be measured accurately (within 0.5 %) at concentrations as low as 0.05 ng/g.

Determination of lower limit of detection

It is common practice in isotope geochemical analysis to report blanks on a "total Pb" basis for a given procedure (i.e., the amount of Pb added by a sample during a procedure). The method LOD (5 pg total Pb) equates to a concentration of 2.5 pg/mL in 2 mL of HNO_3. This limit is derived from Faraday cup sensitivity and signal-to-noise characteristics.

For a 5 pg total Pb blank, and assuming an average atomic weight of 207.2 g/mol, the solution contains 7.27 x 10^9 atoms/mL. At a flow rate of 100 μL/min (one of the standard flow rates for an ST style Elemental Scientific PFA nebulizer), and 2% ion transmission efficiency (the number of ions hitting the detector per second is 1.27 x 10^5), the count rate for ^{208}Pb (the most abundant isotope) is 1.27 x 10^5 ions/s. The relationship between count rate (N/s), current ($I, amps$), and elementary charge ($e = 1.6\ x\ 10 - 19\ coulombs$) is:

$$I = \left(\frac{N}{s}\right) x\ e$$

Using a large resistor (in the case of this study, 10^{11} Ohms), the Faraday cup converts this current to a voltage (V):

$$V = IR$$

The resulting signal for a 2.5 pg/mL solution is approximately 2 x 10^{-3} V (2 millivolts). While the background electronic noise on a Faraday cup is much lower than this value (theoretically 5 x 10^{-6}), the “on-peak” background that includes signals from within the introduction system often exceeds 2 mV of ^{208}Pb, defining our practical limit of detection.

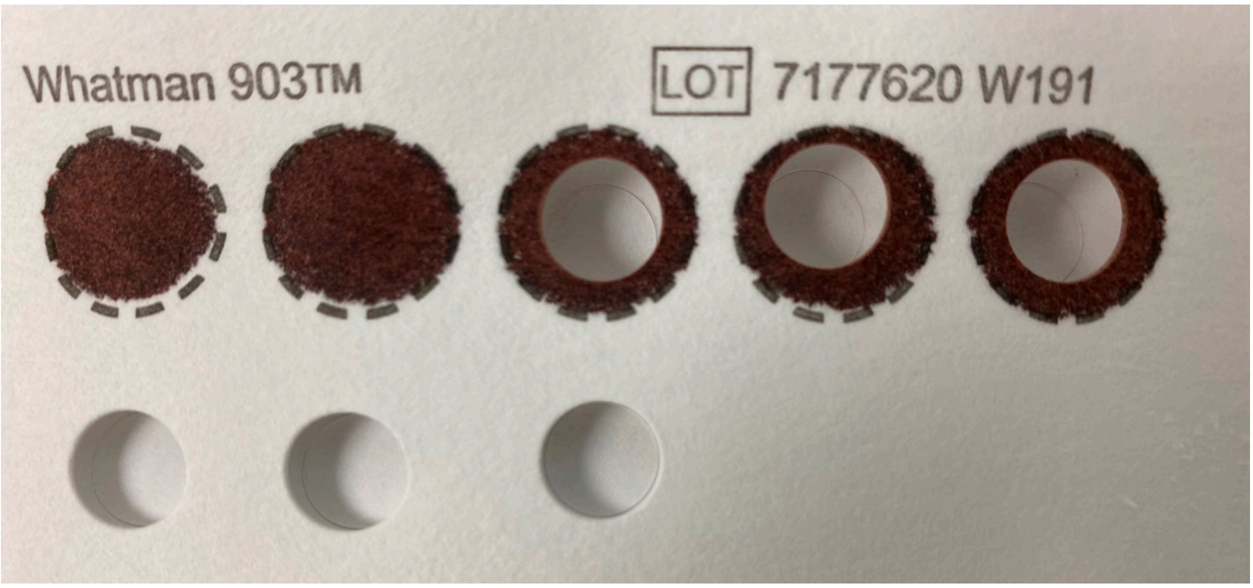

Figure S1. Whatman™ filter paper card commonly used for newborn screening, shown after being spotted with blood and has several 8 mm punches removed for analysis.

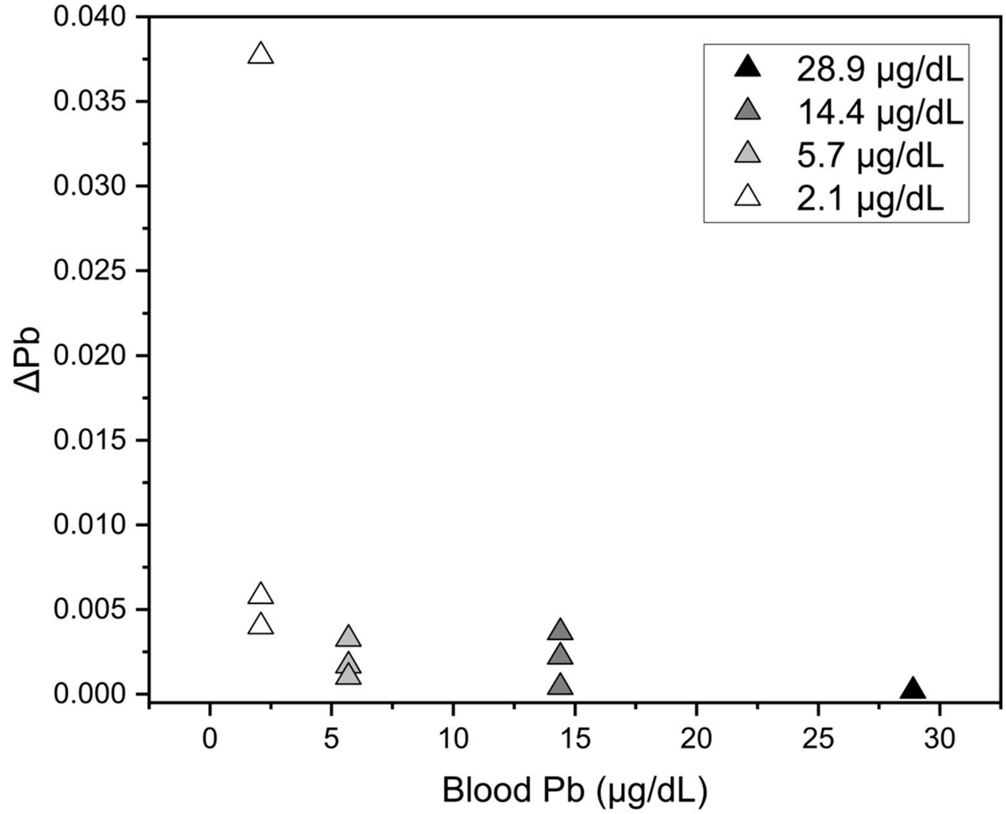

Figure S2. **Method Development Samples.** Comparison of isotopic deviation (ΔPb) versus blood Pb concentration (μg/dL) for spotted blood samples. ΔPb increases as blood Pb concentrations decrease, reflecting greater and more variable blank contributions from the filter paper. Analytical uncertainties of individual data points are smaller than the symbol sizes.

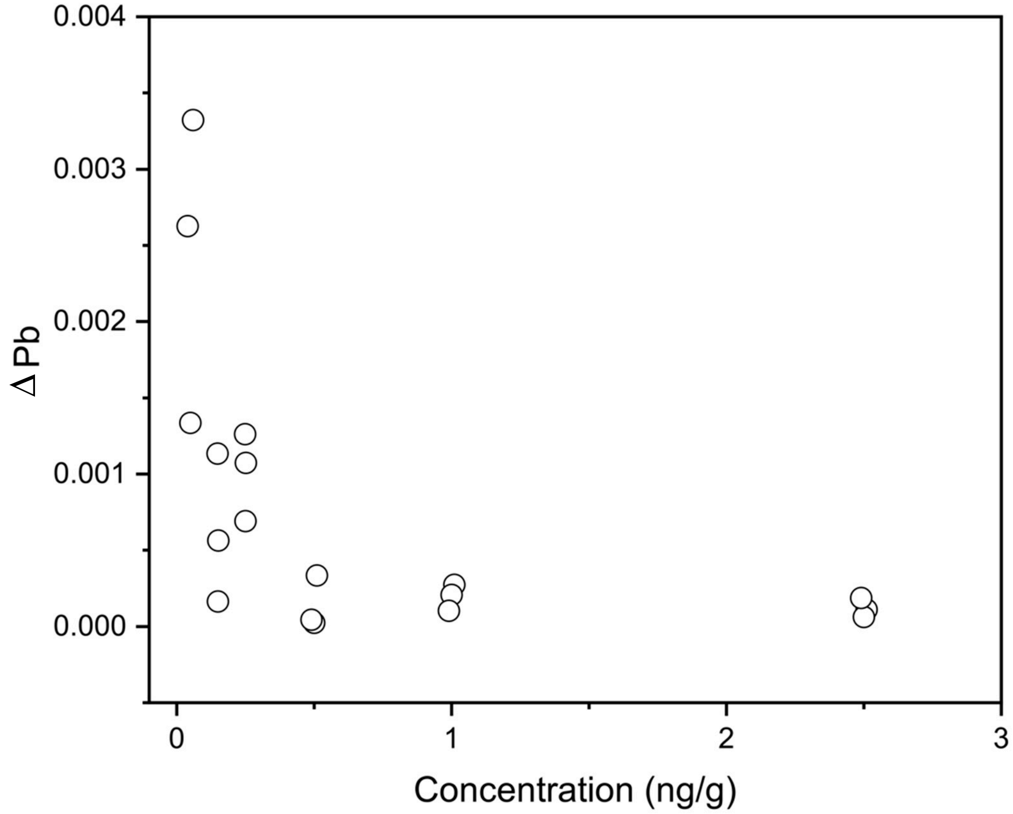

Figure S3. Analytical Validation. Comparison of isotopic deviation (ΔPb) values vs. Pb concentration in NIST SRM 981 standard reference material solutions. The low ΔPb (< 0.004) across all concentrations confirms that DBS deviations are environmental/matrix-based rather than analytical error.

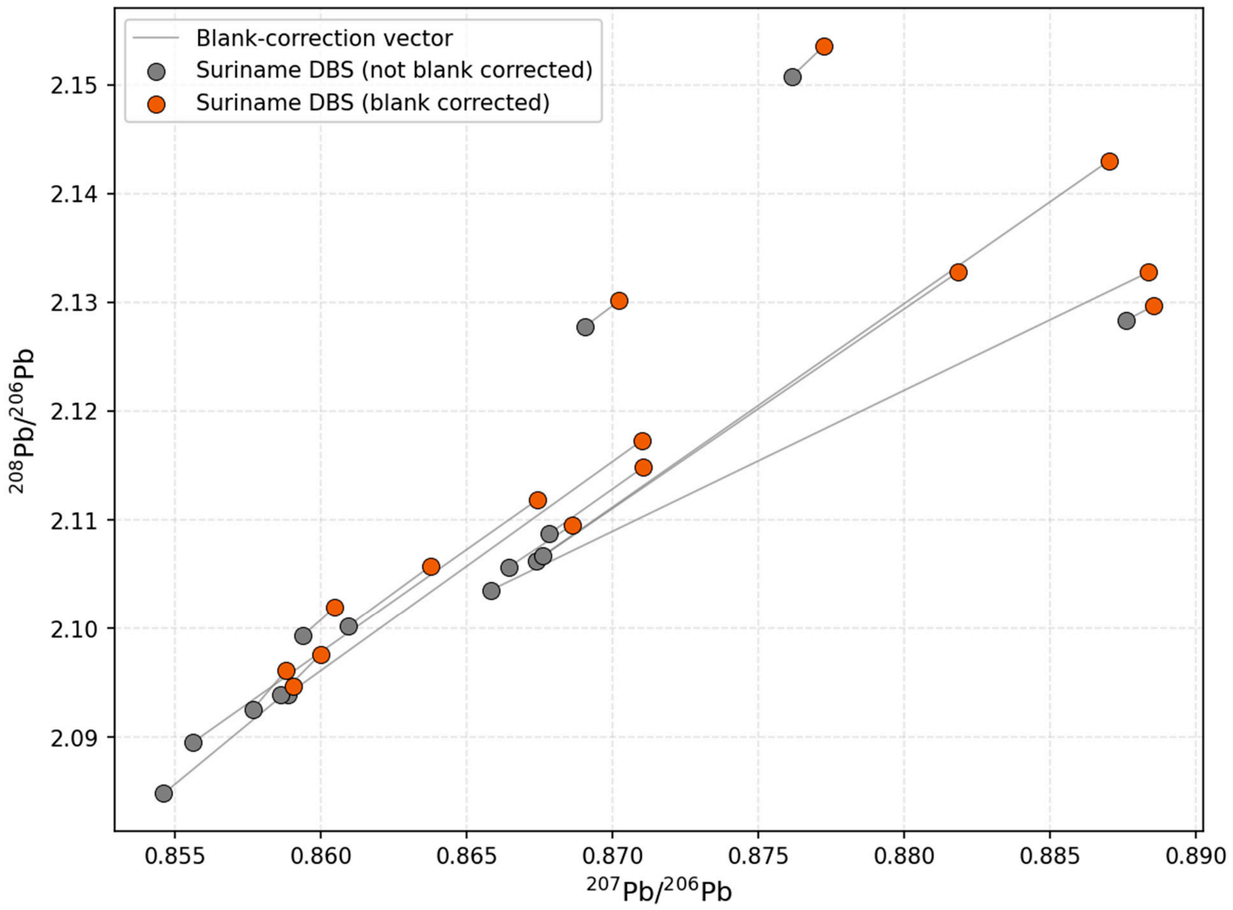

Figure S4. Suriname Data. Plot showing $^{208}Pb/^{206}Pb$ vs. $^{207}Pb/^{206}Pb$ in Suriname dried blood spots before (grey) and after (red) an atom-basis blank correction for filter paper contributions. Each DBS was corrected using its specific adjacent card blank to account for localized filter paper Pb contributions. Faint tie-line (blank-correction vectors) connect paired data points, illustrating the magnitude and direction of the isotopic shift resulting from the recalculation. The consistent upward and rightward trajectory of the vectors confirms that the filter paper background was systematically less radiogenic than the DBS Pb. Vector length reflects the relative influence of the blank atoms on the Pb isotopic composition of the sample.
